# Cambial Activity of *Moringa peregrina* (Forssk.) Fiori in Arid Environments

**DOI:** 10.3389/fpls.2021.760002

**Published:** 2021-11-02

**Authors:** Holger Gärtner, Emad Farahat

**Affiliations:** ^1^Dendrosciences, Forest Dynamics, Swiss Federal Research Institute for Forest, Snow and Landscape Research (WSL), Birmensdorf, Switzerland; ^2^Botany and Microbiology Department, Faculty of Science, Helwan University, Cairo, Egypt

**Keywords:** arid environments, pinning, cambial activity, wood formation, isotopes, vegetative growth

## Abstract

*Moringa peregrina* (Forssk.) Fiori, one of 13 species of the Moringaceae family widely distributed throughout the dry tropics, has the potential to become one of the most economically important medicinal plants in Egypt. However, despite its tolerance for drought and heat, it is also threatened by increasing temperatures and decreasing precipitation. Although the phenophase of this species is well documented, almost nothing is known about its period of cambial activity in desert regions. Ring formation and the general environmental adaptability of trees are affected by the timing of cambial activation. In our study site, we observe a distinct coupling of the development of new green leaves at the onset of vegetative growth in October and the phase of cambial activity (November–January). The onset of cambial activity seems to be related to a drop in temperature in October and the onset of torrential rains in the region. There might even be a short phase between the end of cambial activity and the onset of bud formation without xylem formation, but with photosynthetic activity. If so, we assume that all assimilates are stored as non-structural carbohydrates (NSC) in the parenchyma of the new ring. This potential gap opens new questions regarding the correlation between NSC storage capacity and the timing of remobilization for subsequent ring formation.

## Introduction

The Middle East and northern Africa are expected to experience more severe droughts in the coming decades due to a paralleled increase in temperature and decrease in precipitation resulting from ongoing changes in climate (Dong et al., 2021; El-Geziry, 2021; Tuel et al., 2021). As a consequence, the germination and survival of tree species in the area will be negatively influenced (Copenheaver et al., 2010; Matesanz and Valladares, 2014). In recent years, severe drought events in the Mediterranean basin have suppressed tree growth and increased tree mortality rates (O’Brien et al., 2017; Cramer et al., 2018; Gauquelin et al., 2018). These conditions are especially tough on endangered species in the region; *Moringa peregrina* (Forssk.) Fiori is one such species, although it is known to be drought and heat tolerant (Hegazy et al., 2008). *Moringa peregrina* is one of 13 species of the Moringa family widely distributed in the dry tropics (Olson and Carlquist, 2001). In Egypt, it has the potential to become one of the most economically important medicinal plants (Senthilkumar et al., 2018) on account of the oil produced from its seeds, which is widely used in cosmetics and bioenergy (Vaknin and Mishal, 2017).

Although *M. peregrina* does not produce the same amount of seeds as the cultivated species *Moringa olifeira* Lam., its seeds are bigger and have a higher concentration of oil (Somali et al., 1984; Salaheldeen et al., 2014; Said-Al Ahl et al., 2017). Furthermore, *M. peregrina* is less susceptible to diseases and is therefore favored for more intensive cultivation in the future. The production of interspecies hybrids for the Mediterranean region has been discussed as a way to increase both oil production and disease resistance (Vaknin and Mishal, 2017).

For dendroecological research in tropical dry zones, but also for a potential future cultivation of trees in arid zones, knowledge about the timing of cambial activity and its relation to tree physiology and phenology is crucial (Fritts, 1976; Frankenstein et al., 2005; García-Cervigón et al., 2020; Sánchez-Romero et al., 2021; Schröder et al., 2021; Worbes et al., 2013; Werden et al., 2020). To gain knowledge about the influence of climate on the growth of the species, it is important to analyze correlations between ring width and precipitation and/or temperature. To do this in a reliable way, we need to know the time of cambial activity of the species, to avoid comparing unrelated data (Li et al., 2021; Pandey, 2021). Cambial activity depends on the complex interactions of a number of intrinsic and extrinsic factors (Savidge, 1996; Vieira et al., 2020). However, the influence of temperature (Ren et al., 2018), precipitation (e.g., Rahman et al., 2019), and water potential (e.g., Peters et al., 2021) on cambial activity is high.

The anatomical structures of the rings of deciduous trees are highly variable, especially with regard to their distribution of conducting tissue (vessels) within the ring. The vessel arrangement can be ring-, semi-ring-, or diffuse-porous. Depending on this structure, the onset of cambial activity can also be related to different phenological phases of the tree species (Larson, 1994; Sundberg et al., 2000). In temperate zones, ring-porous species generally start developing their vessels before bud break occurs, whereas diffuse-porous species (such as *M. peregrina*) commonly develop their vessels after leaves are fully developed (Suzuki et al., 1996). However, there are exceptions to this “rule,” as, e.g., Frankenstein et al. (2005) described a contemporaneous cambium activation for diffuse-porous *Acer platanoides* L. and ring-porous *Fraxinus excelsior* L. after bud break.

In the eastern Mediterranean region, there is no constant pattern of cambial activity for, e.g., *Cupressus sempervirens* (Liphschitz et al., 1981) or *Pinus halepensis* (Gindel, 1967). Liphschitz and Lev-Yadun (1986) identified an “adapted Mediterranean type” in the annual rhythm of cambial activity of evergreen sclerophylls and seasonal dimorphics growing along the coasts of the Mediterranean. According to their results, cambial activity in these regions starts in autumn when temperatures drop and the rainy season starts, and remains active until drought stress and high temperatures prevail in early summer. Moreover, there are indications that some tree species show correlations between leaf fall and cambial dormancy (Venugopal and Liangkuwang, 2007).

The cambial activity (in contrast to phenological stages) of desert trees in Egypt has rarely been analyzed in detail (Patel et al., 2014). The few studies available regarding *M. peregrina* (e.g., Hegazy et al., 2008; Keneshloo et al., 2015) have observed that vegetative growth starts in December and lasts until the end of February/beginning of March. The phase of vegetative growth is followed by flowering bud formation until the beginning of April, while flowering starts in late March and last until mid-April. Subsequent fruiting occurs at the end of April and lasts until mid-July. At the end of the fruiting stage (mid-July), seed dispersal occurs until early or mid-August. Trees start shedding their leaves in late June during the fruiting stage. According to Hegazy et al. (2008), dormancy of the shoots began in early or mid-August and lasts until the end of November. Details about the cambial activity and/or xylogenesis of *M. peregrina* are, to our knowledge, not published.

A first detailed attempt to build ring-width chronologies was done by Farahat and Gärtner (2019). Based on literature, the cambial activity (or in this case, the vegetation period) was assumed to be January to March. Results showed positive correlations with the previous year’s April temperature for one site, but negative relationships with April and May–August temperatures of the “current” growing season, i.e., the vegetation period of the year the ring was formed. If these findings are correct, they indicate cambial activity in the respective time period, which would be in accordance with the period of vegetative growth. What remains unclear is why these correlations are positive for April of previous year, but negative for April of the current year, although differences in this month’s temperature are not pronounced. We hypothesize that, if these correlations are not incidental, this might be related to the stage of cambial activity of the respective month. The amount and quality of wood produced, as well as the environmental adaptability of trees, are affected by the timing of cambial reactivation. Because cambial activity requires non-structural carbohydrates (NSC), which are mostly sugars and starch produced during photosynthetically active phases (Hartmann and Trumbore, 2016), we hypothesize that cambial activity and photosynthetic activity coincide in time because it would be beneficial for trees in this harsh environment. Furthermore, the knowledge about the timing of cambial activity, which is controlled by environmental factors, is crucial for all dendroecological reconstructions. The aim of this study is to determine the annual rhythm of cambial activity in *M. peregrina* from the Egyptian Eastern Desert. We decided to apply the pinning method (Kuroda and Shimaji, 1984) to the trees of interest to analyze the onset of growth in detail. Additionally, we applied isotopic measurements of the outermost rings to verify the potential occurrence of ring boundaries, i.e., annual rings, before or at the beginning of our experiment that are real annual boundaries.

## Materials and Methods

### Study Site

**Figure 1:**
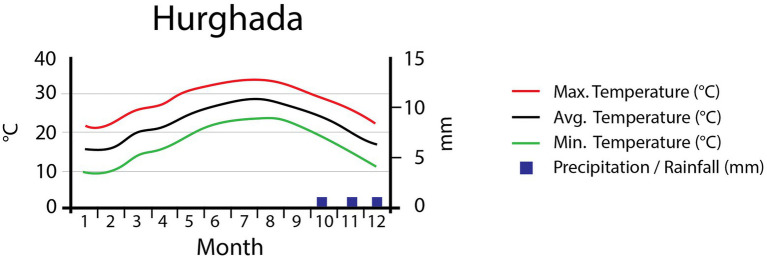
Climate diagram for the study area showing the monthly maximum, mean, and minimum temperatures (°C) and the mean monthly precipitation (mm).

This study was conducted in the mountains of Hurgada, located in the Eastern Desert of Egypt. Classified as a “hot desert” (Köppen-Geiger classification), this region has an annual average temperature of 22.9°C (Figure 1), with January being the coldest month (avg. 15.5°C) and August the hottest (avg. 29.2°C). The total annual precipitation of the area is 3mm/year; no precipitation at all occurs between January and September. The highest precipitation values are reached in October, with a monthly average of 1mm (Data source: Climate data organization, https://en.climate-data.org, accessed on 28 September 2018; Moustafa and Zaghloul, 1996). These precipitation values are broadly representative of the region, but should be treated with care when looking at specific sites. Precipitation frequently occurs as convective rains, causing very localized flash floods. These floods are highly unpredictable, intense, and local in extent (Abd-Elhamid et al., 2018). As a consequence, most of the water is rapidly transported down valley, leaving only a small amount to seep into the ground. Moreover, these sudden flood events bear a high-risk potential to local infrastructure (Dadamouny, 2009; Youssef et al., 2011).

Along the slope of a gorge in the Wadi Om Nefa’a, Hurghada, Red Sea Governorate (27°4'13.07"N, 33°24'29.30"E), 14 *M. peregrina* trees were selected along a 200-m-long altitudinal gradient (600–800m a.s.l.). The area is rocky with stones and rocky fragments on the soil surface. Trees were selected on the basis of their size (biggest individuals) and the “stand density” around the trees. Direct competition of a single tree with neighboring trees for, e.g., water resources could result in an unknown depression of growth, thereby altering the period in which the trees start and end xylem formation. To avoid the influence of any competition for water, we selected trees with no competitors within at least 15m.

### Pinning

Since the aim of this study was to determine the duration (i.e., the start and end) of cambial activity of *M. peregrina* in the area, we decided to apply the pinning method (Kuroda and Shimaji, 1984; Heinrich et al., 2007; Gärtner and Heinrich, 2009) to the selected trees. Although puncher samples (Rossi et al., 2006) would have allowed for an instant analysis of the wood formation at the same moment in time for every individual tree, the stem diameter of the trees (avg. 15cm) at the study site was too small for this method to be an option.

To apply the pinning method, a small needle (diameter<1mm) is pushed into the xylem by penetrating the bark, phloem, and cambium. This puncture destroys the cambial cells in a very small area, i.e., the size of the needle diameter. Because the cambial layer is highly responsive to external disturbances, intact cells around the puncture respond to the damage by producing irregular cells (i.e., callous tissue) and by accelerating growth in the direct vicinity of the destroyed cells. This results in the development of callous tissue around the puncture hole (Kuroda and Shimaji, 1984), while also stopping the formation of new cells at the border of the hole. This results in an area of overgrowth that eventually closes the hole (Seo et al., 2007). As an immediate reaction to disturbance, radially flattened cells extending a few cell rows laterally from the wound form a “pinning line,” indicating the moment of cambial destruction within the ring.

Although the site was not easy to reach, all trees were pinned six times in 2019 (January 27th, March 1st, April 11th, June 27th, August 20th, and October 27th). Each time, photos were taken of the area and the trees to document the phenological stages of the trees at the time of pinning.

### Anatomical Analyses

On January 8th, 2020, the trees were felled and their stem sections were taken to the laboratory for further analysis. Discs were cut out of the stem sections covering one pinning hole each resulting in six separate disks per tree. The disks were sanded down to approximately 1mm above the pinning hole. Then, a rectangular piece containing the bark, phloem, cambium, and the outermost rings was split out of each disc with the pinning hole in the center.

These samples were fixed in a microtome (Gärtner et al., 2015) and micro-sections (thickness: 15μm) were cut. For each disk containing a pinning hole, four sections were cut and analyzed to ensure that at least one section showed the anatomical structure around the center of the pinning hole. The sections were then double-stained with Safranin (which turns lignified structures red) and Astra blue (which turns unlignified structures blue) and embedded in Canada Balsam on glass slides following standard protocols (Gärtner and Schweingruber, 2013).

The slides were then scanned using a slide scanner (Axio Scan Z1, Zeiss, Germany) at 100× magnification. The digital images were analyzed visually by determining the pinning line and the number of cell layers developed in the time between pinning was applied and the sampling of the trees.

### Isotope Analysis of Wood Rings From 2019 and 2020

To verify our assumption that the xylem formed before and after the pinning experiment represent separate rings, we took stem disks from an additional six trees to analyze the tree-ring cellulose δ^13^C in both rings. After separating wood blocks from each of the two rings, each wood sample was placed in an Eppendorf vial (2ml) for milling using an ultracentrifugation mill (Retsch mill ZM200). Samples were then transferred to sealed Teflon bags. To extract α-cellulose, all bags were put in Erlenmeyer flasks (250ml) and the modified Jayme-Wise method was applied (Wieloch et al., 2011). To extract resin, fatty acids, etheric oils, and hemicellulose we added NaOH (7% solution) to the Teflon bags. Then the bags were heated in a water bath at 60°C for 2h. After repeating this step twice, they were rinsed with distilled water at 60°C three times. To extract lignin a solution of 7% NaClO_2_ was added; the bags were then heated again to 60°C for 40h. This process was repeated three times every 10h. The samples were then rinsed again three times with boiling distilled water, squeezed to remove excess water, and placed in an oven at 60°C for at least 4h. The yield for all samples was approximately 30–45% cellulose. For each sample, 0.9–1.0mg of cellulose was then packed into silver capsules for isotopic analysis. Cellulose was pyrolyzed using a High-Temperature Elemental Analyzer (Pyrocube, Elementar, Hanau, Germany); stable carbon (δ^13^C) isotope composition was measured using a Continuous Flow Isotopic Ratio Mass Spectrometer (IRMS Delta XP Advantage, Thermo Fisher Scientific, Bremen, Germany) *via* a variable open split interface (Conflo III, Thermo Fisher Scientific, Bremen, Germany; Woodley et al., 2012). The analytical errors (standard deviations) of the isotope measurements were less than 0.3‰ for δ^13^C. The δ^13^C isotopic composition is presented as the deviation (δ) of the sample from the international standard for δ^13^C (VPDB). The mean values of δ^13^C for both rings were subjected to paired sample *t*-test analysis.

## Results and Discussion

**Figure 2:**
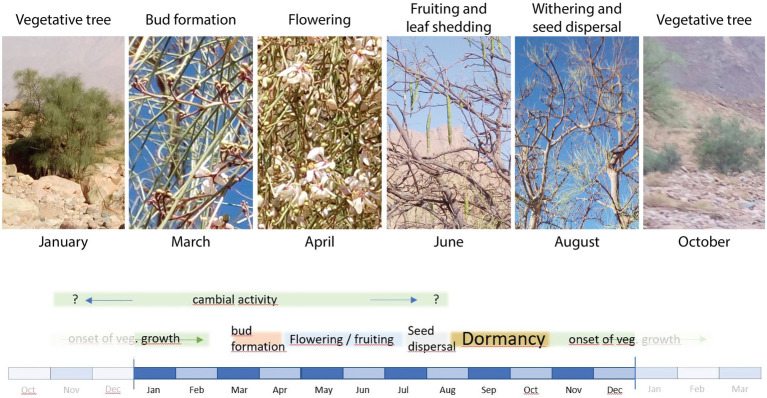
Images indicating phenological stages of *Moringa peregrina* at the time of pinning. Photos were taken on January 27th, March 1st, April 11th, June 27th, August 20th, and October 27th, 2019. The lower scheme illustrates the onset and duration of these stages as well as the potential period of cambial activity based on the literature published so far.

The phenological stages of *M. peregrina* trees observed at the study site were roughly in accordance with previously published descriptions of the species (Figure 2).

From our field observations, *M. peregrina* is deciduous and began to drop its leaves at the end of April. In this case, the small leaflets formed around the leaf axis dropped while the green axis remained on the branch. In early March, bud formation was already active and flowering began, while the flowering stage lasted until at least mid-April. Fruiting was active and some fruits had already ripened by June. By August, all fruits had fallen, i.e., seed dispersal was already over. At this point, the withering phase was in full progress and the leaflets were falling off. This indicates that *M. peregrina* goes dormant by mid- to late August. In contrast to the observations of Hegazy et al. (2008), we found that new leaves had started to develop at the time of the last pinning in late October. Thus, in our study area, the vegetative phase starts in October, which is when flooding begins, and reaches a peak during January and early February. Consequently, trees in the area are not dormant until the end of November, beginning of December as reported by Hegazy et al. (2008). According to Keneshloo et al. (2015), growth patterns of *M. peregrina* vary depending on annual temperature, fluctuations in precipitation, and drought. They also note that although environmental conditions vary in different areas, a minimum temperature of 24.7°C during the time of tree growth is required for seed ripening. The remaining question is, does this period of seed ripening also correspond with cambial activity?

**Figure 3:**
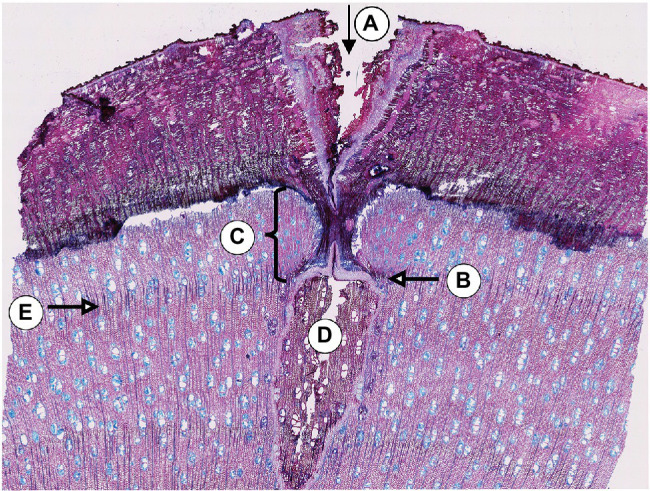
Micro-section indicating the pinning area of the third pinning event in tree no. 1 (April 11th, 2019). A: Pinning hole in the bark; B: Callous tissue formed as a reaction of the irritated cambial cells surrounding the pinning hole; C: Amount of xylem produced after pinning until the felling date; D: Pinning hole in the xylem that was already formed at the time of pinning; E: Ring boundary of the ring formed before pinning.

**Figure 4:**
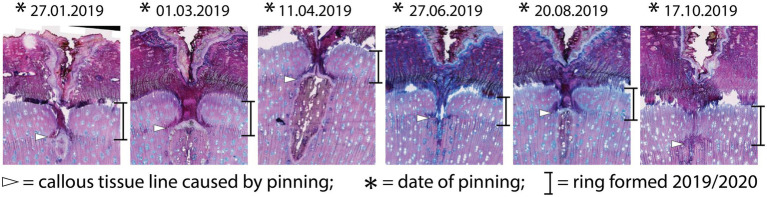
Micro-sections indicating the growth reaction of the trees to the pinning experiment. All sections show the same reaction, regardless of the time of year the pinning was done.

A visual analysis of the pinning micro-sections revealed that all trees showed anatomical reactions to the disturbance caused by the puncture of the cambium where the needle penetrated the cambial layer (Figure 3). All sections cut through the center of the pinning hole showed the typical pinning hole in the bark and in the xylem formed before pinning. They also show the callous tissue that the irritated cambium produced around the wound. Immediately after the formation of this callous tissue, the number of cells surrounding the wound increased (Figure 3C) to help close the hole and prevent pathogens from penetrating the open wound area. This growth reaction is a particularly effective part of the defense system of trees and shrubs (Gärtner and Heinrich, 2013). What is interesting about the growth reactions found in all sections is that they all show the same reaction. There is no difference in the reactions to the pinnings exerted between January and October (Figure 4).

**Figure 5:**
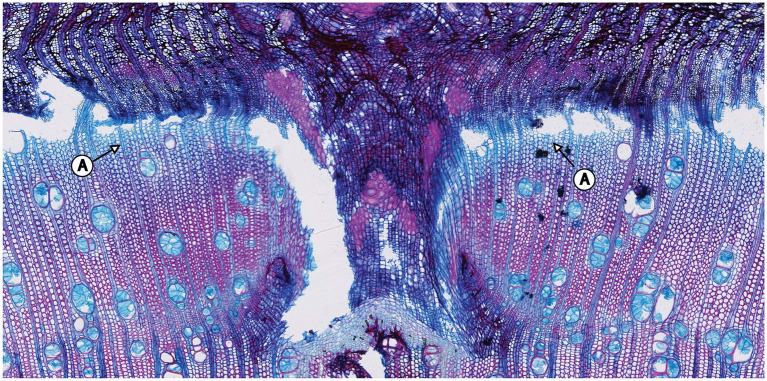
Pinning area of the first pinning exerted to tree no. 5. A: The sample was stained in Safranin (red) and astra blue. Nevertheless, the outermost cells of the ring are blue in color, indicating that they have not yet lignified.

The samples of all trees showing the first pinning exerted on January 27th, 2019 show callous tissue (and/or a callous line) as the first new cells of the outermost ring visible in the section. Because no cells of the ring existing before pinning were irritated and for this reason do not show any sign of disturbance, this ring was fully developed and lignified before the first pinning. Thus, the pinning was applied in the dormant phase of the cambium because cambial activity must have ended before January 27th, 2019. The new ring was therefore definitely formed after the first pinning. An analysis of the micro-sections representing the pinnings applied after the first pinning indicate similar results for all trees. As shown in Figure 4, all micro-sections show a ring formed after the destruction of the cambium caused by the pinning. There is no difference in the characteristics of the single growth reactions to the wounding of the cambium. The timing and intensity of the onset of callous tissue was similar in all samples, so the cambium of the trees was inactive during the entire pinning period. The new ring started growing *after* the last pinning was exerted (i.e., after October 17th, 2019). Additionally, the outermost cells of the outermost ring in all trees appeared blue after staining, indicating that the secondary walls of these cells were thickened but not lignified (Figure 5).

The quantity of blue cells varies in the samples within trees but also between individual trees. Nevertheless, this feature is restricted to a few outermost cell layers, indicating that the ring-forming process was ongoing but close to its end at the time of felling.

Given that the cambium was not active at the time of the first pinning (January 27th, 2019), we conclude that ring formation (cambial activity as well as lignification) ends around mid- to late January. The phase of cambial activity in *M. peregrina* trees in our study area starts at the end of October/beginning of November and lasts until mid- to late January. Based on the timing of the growth reactions caused by the different pinnings, we assume that the activation of cambial activity in *M. peregrina* is, in accordance with the findings of Liphschitz and Lev-Yadun (1986), caused by decreasing temperatures in October and the availability of water resources in form of subsequently occurring flood events. Nevertheless, the duration of cambial activity is much shorter than expected.

**Figure 6:**
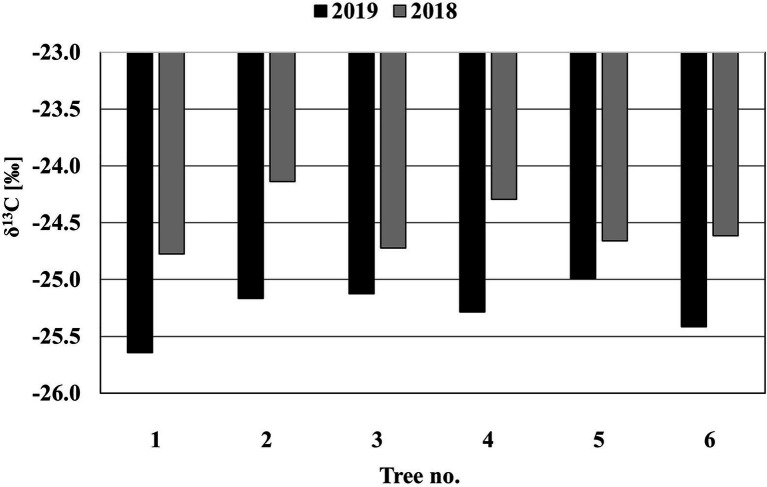
The δ^13^C isotope results of the two rings formed before (2018) and after (2019) the pinnings in the stems of M.

The results of the analysis of the δ^13^C isotopes formed in the two rings before and after the pinnings (Figure 6) show that the mean values of δ^13^C in the ring formed before the pinning (2018; −24.5±0.26) and after the pinning (2019; −25.3±0.23) are significantly different [*t*(6)=−6.07, *p*=0.002].

This confirms our hypothesis that the xylem formed after the final pinning represents a newly formed ring that developed under different moisture conditions than the one that existed when the tree was last pinned (i.e., in October).

**Figure 7:**
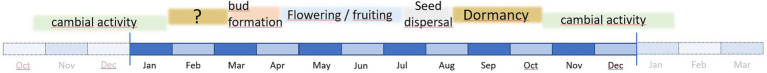
The scheme illustrates the onset and duration of the phenological stages (compare to Figure 2) and the period of cambial activity as determined by the results of the pinning experiment. The period marked with “?” most likely refers to bud formation, although this requires further studies on phenology of the species.

Our observations indicate that new leaves begin developing in mid-October in the study area, heralding the onset of vegetative growth. Cambial activity and thus the formation of a new ring obviously follows the start of vegetative growth (Figure 7). We therefore assume that ring formation is supported by assimilates produced by the new leaves, not just by NSC remobilized from parenchyma cells from the previous year’s growth. Since ring formation is finalized by the end of January, the assimilates produced during the peak of vegetative growth are directed to the formation of flowering buds, flowers, and seeds (Kikuzawa and Lechowicz, 2011; Miyazaki, 2013). However, many studies show that carbon reserves do not decline after fruiting in species in which the modules autonomously allocate current photosynthates for fruiting (Miyazaki, 2013).

Our study indicates that there are two priorities for *M. peregrina* trees in the study area: (1) ring formation right after the beginning of vegetative growth in October, depending on the availability of new assimilates and NSC stored in the previous year’s ring, and (2) the formation of flowering buds after the period of ring formation (cambial activity). It is likely that all assimilates produced after the end of cambial activity are directed toward bud formation, flowering, and seed production. It remains an open question as to whether there is a short period between the end of cambial activity and the onset of bud formation, and whether assimilates are fully stored as NSC in the rays or if these phases have a continuous transition. In the latter case, only surplus assimilates can be stored as NSC in the parenchyma cells during the phase of vegetative growth.

If there is a short phase during which all assimilates are stored as NSC, these reserves could support tree growth and metabolism in times when current photosynthates are insufficient, which needs to be analyzed in detail in future studies. A tree’s capacity to store NSC is a crucial measure for its resilience in times of stress (Richardson et al., 2013). It is for this reason that in-depth analyses of plant physiological processes are needed to fully understand how environmental factors influence the growth of *M. peregrina* in our study area and elsewhere.

## Conclusion

In summary, we were able to gain new insights regarding cambial activity in *M. peregrina*. The consistent response to pinning from January to October indicates that the tree is dormant during this period. The onset of the formation of the current year ring begins toward the end of October, early November. Cambial activity ends at the end of January; for this reason, rings are dated based on the year in which growth began. *Moringa peregrina* trees form one ring in an annual cycle that spans two calendar years. The annularity of the rings is supported by the fact, that the wood of each ring has a significantly different δ^13^C isotope value. Finally, the formation of growth rings in *M. peregrina* appears to be triggered by a decline in temperature at the end of summer and the availability of water carried by the infrequent flash floods that occur in October and November in the study area.

## Data Availability Statement

The raw data supporting the conclusions of this article will be made available by the authors, without undue reservation.

## Author Contributions

HG designed the study. EF conducted the field experiment and sampling. HG and EF analyzed and discussed the data, and wrote the manuscript. All authors contributed to the article and approved the submitted version.

## Funding

Funding for this study was provided by the Swiss National Science Foundation (SNSF; grant no. IZSEZ0_192144/1, 2019).

## Conflict of Interest

The authors declare that the research was conducted in the absence of any commercial or financial relationships that could be construed as a potential conflict of interest.

## Publisher’s Note

All claims expressed in this article are solely those of the authors and do not necessarily represent those of their affiliated organizations, or those of the publisher, the editors and the reviewers. Any product that may be evaluated in this article, or claim that may be made by its manufacturer, is not guaranteed or endorsed by the publisher.
